# The Recent Trial for Rape in Montreal

**Published:** 1859-01

**Authors:** 


*The recent Treal for Rape at Montreal.*—In a late number, we took occasion to refer to the trial of a dentist in Montreal, for an alleged criminal assault upon a female patient whom he had rendered insensible by the inhalation of chloroform. The editors of the Montreal Chronicle, while they agree with us in the opinion that the defendant was unjustly condemned, think that an important element in the settlement of the question would be the nature of the anæsthetic agent employed, because, according to them, sulphuric ether is much more likely than chloroform to cause erotic ideas, when inhaled. We believe this opinion to be wholly unfounded. It is not common, we presume, for such effects to follow the administration of either agent, but they do sometimes unquestionably occur, and as often with chloroform as with ether. The fact is, that the plaintiff in this case, as happened in the celebrated case of Dr. Beale, of Philadelphia, was menstruating at the time. The sexual functions were consequently in a state of excitement, and the administration of any stimulant, even a couple of glasses of champaigne wine, would have been likely to create erotic ideas, and to vividly impress the patient with the belief of their reality. The instances of such effects from chloroform are perfectly well authenticated, and one was testified to by a medical genteman during the trial.

We take this opportunity of again protesting against the injustice of allowing the testimony of a person concerning facts which took place while he or she was in a state of complete or partial insensibility, unless corroborated by other evidence, to have any great weight in a case like this. Whose life or reputation is safe, if a patient can so easily swear it away? It was not even established that any rape had been committed at all, any more than in the Philadelphia case to which we previously alluded, before the trial took place. We cannot forbear also commenting upon the extraordinary verdict rendered at the Montreal trial. If the defendant were "guilty of an attempt to commit a rape," upon what grounds was he entitled to a "recommendation to mercy"? What circumstances can palliate such an attempt, especially in a case like the present, where the crime would be a most atrocious violation of confidence? Either the defendant was guilty or not guilty, there could be no other alternative; and, if guilty, he ought to be subjected to the heaviest penalty prescribed by the law.—*Boston Med. & Sur. Journal.*

